# Transitory ischemic attack associated with a rare fenestration of the cervical segment of the internal carotid artery: a case report

**DOI:** 10.1186/s13256-021-03227-0

**Published:** 2022-01-15

**Authors:** Christian Nasel, Angelina Poetsch, Cornelia Brunner, Ewald Moser

**Affiliations:** 1 Department of Radiology - University Hospital Tulln, Karl Landsteiner University of Health Sciences, Alter Ziegelweg 10, A, 3430 Tulln, Austria; 2 Department of Medical Imaging and Image-guided Therapy, Medical University of Vienna, Vienna, Austria; 3 Center for Medical Physics and Biomedical Engineering, Medical University of Vienna, Vienna, Austria; 4 Department of Neurology - University Hospital Tulln, Karl Landsteiner University of Health Sciences, Tulln, Austria

**Keywords:** Carotid artery disease, Stroke, Neurovascular interventions, Magnetic resonance imaging

## Abstract

**Background:**

Fenestration of the cervical segment of the internal carotid artery is a very rare finding, and its origin is still not fully understood. Explanations of its genesis range from dissections leading to the fenestration to the more common interpretation as a developmental vascular variant. However, most reported cases were symptomatic and presented with dissections, where even endovascular treatment of the fenestration of the cervical segment of the internal carotid artery became necessary. Here we report a case of a fenestration of the cervical segment of the internal carotid artery suffering a transitory ischemic attack and local pain in absence of any sign of dissection.

**Case presentation:**

A 62-year-old Caucasian male patient was admitted to our institution because of an episode of amaurosis fugax, initially accompanied with headache. Magnetic resonance imaging revealed an intact fenestration of the cervical segment of the internal carotid artery on the symptomatic side. With antiplatelet therapy, all symptoms vanished within 2 months of the initial event.

**Conclusions:**

Our findings support the interpretation of a fenestration of the cervical segment of the internal carotid artery as a developmental vascular variant, but also suggest a substantial risk for dissection and ischemic stroke. Even in case of an accidental finding, clinicians should be aware of this. At least in this case, antiplatelet therapy seemed beneficial.

## Background

Fenestration in the cervical segment of the internal carotid artery (fcICA) is a very rare finding, with only few cases reported so far [1–4]. The possible embryonic mechanism is not fully understood, causing doubt about the developmental nature of this variant [3]. However, independent from the explanations of its origin, most authors agree that a fcICA potentially predisposes to vessel dissection and ischemic stroke [2–4]. Here, we report a patient presenting with a transitory ischemic attack (TIA) related to an ipsilateral fcICA, who might have profited from antiplatelet therapy.

## Case presentation

A 62-year-old Caucasian male was admitted to the emergency outpatient clinic of another hospital because of an episode of amaurosis fugax and headache on the right side with pain projecting into the right skull base. Additionally, the patient experienced a transient weakness of the lower limbs. At arrival, most of the symptoms had already vanished. The patient’s clinical history was otherwise unremarkable, accept for a borderline hypertonia and an ongoing vascular therapy initiated by the general practitioner of 100 mg acetylsalicylic acid, for cardiac reasons. Cranial computed tomography (CT) at admission was inconspicuous for hemorrhage and ischemia.

Magnetic resonance imaging (MRI) performed 4 days after the initial event, including diffusion-weighted MRI, also showed regular findings, and MR angiography (MRA) of the cerebral as well as ultrasound of the supra-aortic vessels revealed no stenotic vascular process.

**Fig. 1:**
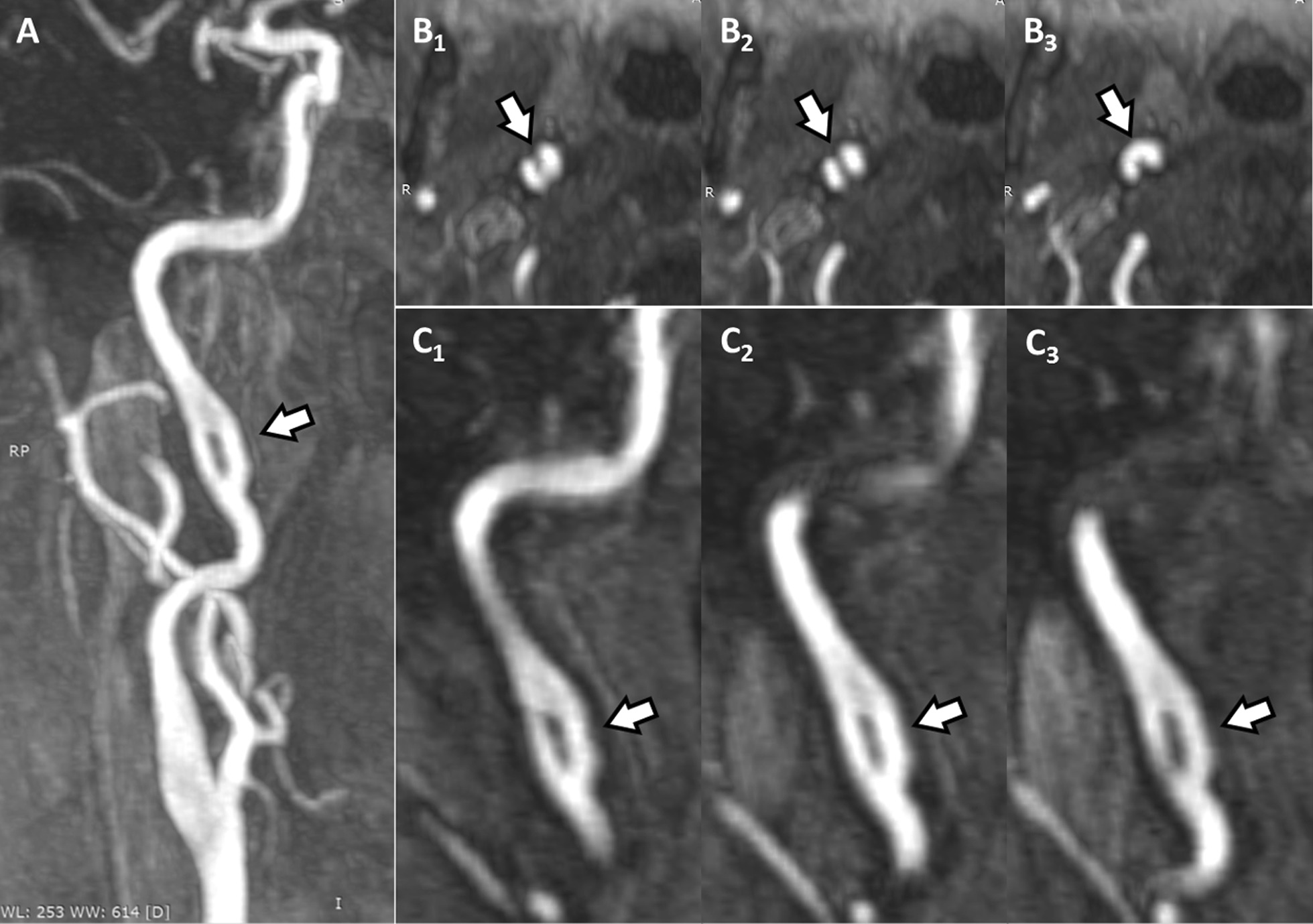
Magnetic resonance angiography performed 2 weeks after the initial event revealed a fenestration of the cervical ICA (**A** white arrow). Multiple transversal sections showed regular filling of the fcICA with contrast medium in both lumina (**B1**–**3*** white arrows*), where paracoronal reformations using a maximum intensity technique showed that both lumina were comparably well developed (**C1**–**3*** white arrows*)

**Fig. 2:**
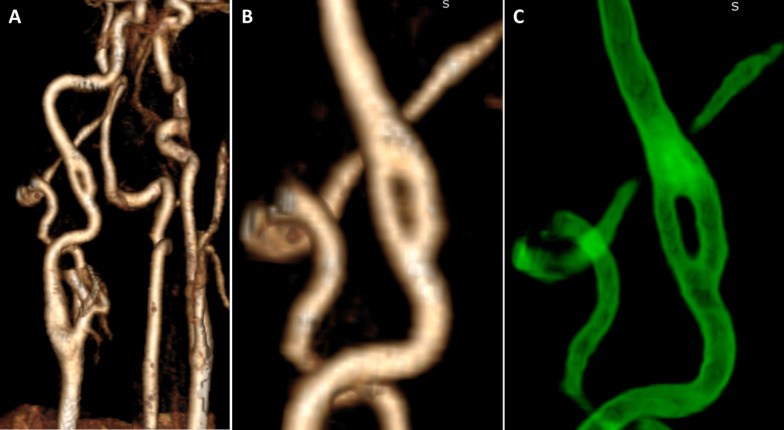
Volume rendering of magnetic resonance angiography data (**A**, **B**) and use of common edge enhancement tools (**C**) did not show any sign of a dissection or any other severe degradation of the fcICA vessel walls

**Fig. 3:**
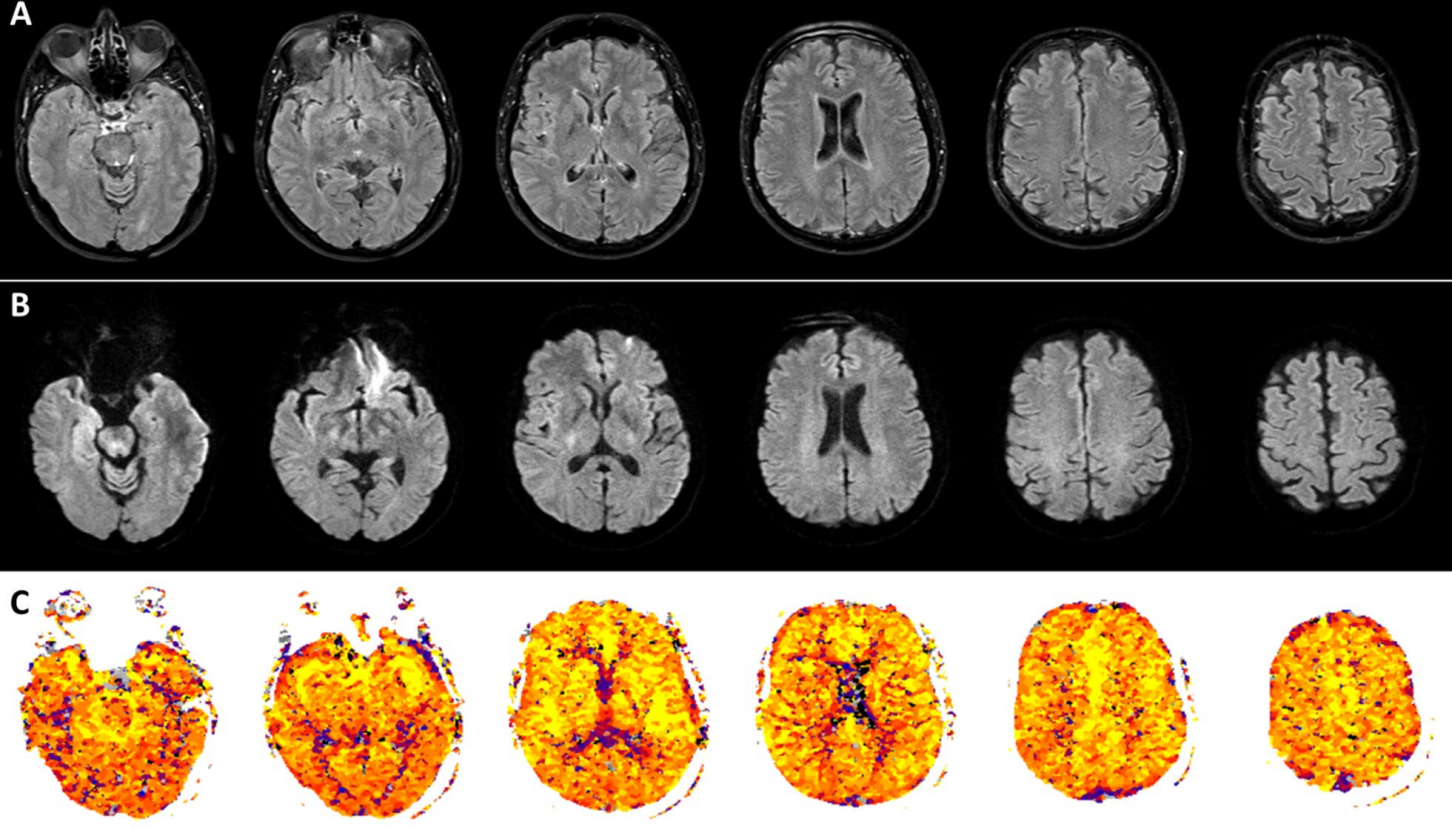
Multiparametric magnetic resonance imaging of the brain, including axial fluid-attenuated inversion recovery imaging (**A** Second long TE echo of a protoneus sequence), dual *b* diffusion-weighted magnetic resonance imaging (**B**: *b* = 1000 s/mm^2^ echo) and perfusion MRI (**C** standardized time-to-peak perfusion map: yellow, orange, and red colors indicate regular perfusion) ischemic brain injury and hemorrhage were ruled out

Due to a faint persisting headache on the right side, the patient was sent to our endovascular outpatient clinic at the radiological department 2 weeks later, where multiparametric MRI of the extra- and intracranial cerebrovascular system revealed a fcICA on the right side (Fig. 1). The fcICA lay about 20 mm underneath the skull base and did not give rise to any other aberrant vessel. It was rather short (approximately 16 mm), with both lumina comparably well developed and regularly perfused. A close workup of the MRA data showed no signs of an fcICA dissection (Fig. 2). Brain imaging was still negative for ischemia or hemorrhage (Fig. 3). Supposing a transitory ischemic attack, probably triggered by the ipsilateral fcICA, the antiplatelet therapy was continued. The patient completely recovered within 2 months of the initial event.

## Discussion and conclusion

Worldwide, only few cases of a fcICA are known. The apparently self-evident developmental nature of the fcICA was doubted because, in most cases, signs of a dissection of the involved vessel walls were observed [5]. While most authors considered this dissection as a complication of the fcICA, others hypothesized that the observed fenestration could result from the dissection of a previously regularly developed vessel [3].

Following the developmental embryonic interpretation, an fcICA could be imagined as an abortive variant of a complete internal carotid artery (ICA) duplication, which represents another very rare vessel anomaly [1, 6]. Likewise, according to the dissection-theory, an fcICA has to be clearly separated from the complete ICA duplication variant, because the embryonic origin of the ICA duplication is well explained by the simultaneous development of a normal ICA together with another aberrant vessel that forms from material of the first and second primitive aortic arches [3, 6–8]. In this patient, the fcICA exhibited two comparably well developed and perfused lumina, with regular wall structures without any signs of dissection. Interpreting one lumen as the regular and the other as the aberrant one, from the morphological point of view, this case does not support the theory of a dissection eventually forming the cervical ICA fenestration, but rather suggests an embryonic origin of the fcICA.

Independently of its supposed origin, it is reasonable to assume that the clinical symptoms in our patient were indeed inflicted by the fcICA. The spectrum of symptoms reported in literature ranges from ipsilateral stroke to cervical pain, where these symptoms were nearly always found in the presence of a dissection or even an occlusion of the fcICA [1–4]. Despite the symptoms encountered in this case, the fcICA itself was found morphologically fully intact. Nevertheless, it is conceivable that the pain experienced by the presented patient was probably triggered by an incipient, not yet detectable, pathological wall degeneration, rendering the preexisting fcICA prone to a later dissection. In the same way, the transient amaurosis of the right eye in this patient could have evolved from thrombotic vessel alterations related to the proposed wall degenerations. The known borderline hypertonia of our patient could then have acted as a risk factor for the vessel wall affection, as a continual increase of the systolic arterial blood pressure could potentially increase regional flow turbulence, with a higher shear stress at the luminal bifurcation of the fcICA. Considering the strong correlation of an amaurosis fugax with stenotic ICA disease, a conjunction between symptoms detected in this patient and the postulated incipient degeneration of the fcICA seems, at least, possible. In this context, this patient could have profited from the already ongoing antiplatelet therapy.

However, an fcICA obviously carries a substantial risk for dissection and stroke and should, therefore, be kept under observation [1, 3–5]. As mentioned, our patient could have profited from the ongoing antiplatelet therapy of 100 mg acetylsalicylic acid, where symptoms regressed completely over time. Unfortunately, due to its extremely low incidence, a conclusive prospective study concerning prophylactic medical treatment of a symptomatic fcICA is not available. So far, only 13 cases have been described in detail in the literature [5]. One larger case series on fenestrations of cerebral arteries in general also found direct affections of the internal carotid artery (without differentiating real cervical from the more frequent intracranial ones) in less than 15% [9]. Though general treatment guidelines for vascular dissections are available, recommendations about dealing with an otherwise intact fcICA cannot be given at the moment. However, in case of an acute fcICA occlusion with impending stroke, endovascular treatment with stenting seems to be an option [4].

As our patient fully recovered after the initial event, we had to refrain from intraarterial digital subtraction angiography (iaDSA) to further assess the fcICA, which limits our exploration. With respect to the exposure to radiation for follow-up examinations, iaDSA and CT angiography, which both clearly depict an fcICA, should be considered carefully. Since MRI and MRA, as in our analysis, were found reliable to detect a dissection, which is probably the main cause of fcICA-related symptoms [10], MRI might be the best choice for follow-up. Ultrasound may be an excellent choice as well, but examination of a vascular anomaly near the skull base is challenging, if not impossible.

In agreement with the few reported cases so far, our case supports the classification of an fcICA as an arterial developmental anomaly that carries a substantial risk of dissection and stroke [5]. Like in our patient, a TIA may occur independently from a detectable dissection, but follow-up examinations of an fcICA are recommended, as symptoms may potentially precede a dissection.

## Data Availability

Not applicable. All data (images) generated or analyzed during this study are included in this published article.

## Abbreviations

fcICA: Fenestration of the cervical segment of the internal carotid artery
TIA: Transitory ischemic attack
CT: Computed tomography
MRI: Magnetic resonance imaging
MRA: Magnetic resonance angiography
iaDSA: Intraarterial digital subtraction angiography
