# Correction to: Transitory ischemic attack associated with a rare fenestration of the cervical segment of the internal carotid artery: a case report

**DOI:** 10.1186/s13256-022-03341-7

**Published:** 2022-02-28

**Authors:** Christian Nasel, Angelina Poetsch, Cornelia Brunner, Ewald Moser

**Affiliations:** 1 Department of Radiology, University Hospital Tulln, Karl Landsteiner University of Health Sciences, Alter Ziegelweg 10, A, 3430 Tulln, Austria; 2 Department of Medical Imaging and Image -guided Therapy, Medical University of Vienna, Vienna, Austria; 3 Center for Medical Physics and Biomedical Engineering, Medical University of Vienna, Vienna, Austria; 4 Department of Neurology, University Hospital Tulln, Karl Landsteiner University of Health Sciences, Tulln, Austria

## Correction to: J Med Case Reports (2022) 16:13 https://doi.org/10.1186/s13256-021-03227-0

Following publication of the original article [1], the authors reported errors in the contributing author names. The given name and family name were erroneously transposed.

The incorrect author names reads:

Nasel Christian

Poetsch Angelina

Brunner Cornelia

Moser Ewald

The correct author names should read:

Christian Nasel

Angelina Poetsch

Cornelia Brunner

Ewald Moser

The author group has been updated above and the original article [1] has been corrected.
